# Exogenous Molecular Probes for Targeted Imaging in Cancer: Focus on Multi-modal Imaging

**DOI:** 10.3390/cancers2021251

**Published:** 2010-06-11

**Authors:** Bishnu P. Joshi, Thomas D. Wang

**Affiliations:** 1 Division of Gastroenterology, Department of Medicine, University of Michigan, School of Medicine, 109 Zina Pitcher Place, BSRB 1722, Ann Arbor, MI 48109, USA; 2 Department of Biomedical Engineering, University of Michigan, Ann Arbor, MI 48109, USA

**Keywords:** molecular probes, contrast agents, affinity ligands, peptides, antibodies, nanoparticles, radioligands, imaging, cancer, optics, PET/SPECT, MRI, US

## Abstract

Cancer is one of the major causes of mortality and morbidity in our healthcare system. Molecular imaging is an emerging methodology for the early detection of cancer, guidance of therapy, and monitoring of response. The development of new instruments and exogenous molecular probes that can be labeled for multi-modality imaging is critical to this process. Today, molecular imaging is at a crossroad, and new targeted imaging agents are expected to broadly expand our ability to detect and manage cancer. This integrated imaging strategy will permit clinicians to not only localize lesions within the body but also to manage their therapy by visualizing the expression and activity of specific molecules. This information is expected to have a major impact on drug development and understanding of basic cancer biology. At this time, a number of molecular probes have been developed by conjugating various labels to affinity ligands for targeting in different imaging modalities. This review will describe the current status of exogenous molecular probes for optical, scintigraphic, MRI and ultrasound imaging platforms. Furthermore, we will also shed light on how these techniques can be used synergistically in multi-modal platforms and how these techniques are being employed in current research.

## 1. Introduction

Significant progress has been made in the field of *in vivo* molecular imaging of cancer due to recent advances in molecular biology, coupled with the rapid development of innovations in imaging instrumentation and probe chemistry. Several modalities have been utilized for targeted imaging in cancer such as ultrasound (US), computed tomography (CT), scintigraphic (PET/SPECT), magnetic resonance imaging (MRI), and optics [1,2,3,4,5,6]. These techniques have played a significant role in all aspects of cancer, including diagnosis, staging, risk stratification, planning and guidance of therapy, and chemoprevention. The development of novel contrast agents is critical to the use of different imaging modalities. For example, various radioisotopes, such as ^123^I, ^99m^Tc, ^64^Cu, ^111^In, ^11^C, ^13^N, ^15^O and ^18^F for SPECT/PET, supermagnetic or paramagnetic metals for MRI, microbubbles for US, and various visible and near-infrared (NIR) dyes for optical imaging have been developed. Most of these compounds, however, are non-targeted agents that provide non-specific contrast. Anyway, the cutting edge of targeted *in vivo* imaging is represented by many examples of nuclear medicine applications.

Molecular imaging has raised the level of interest for the detection and management of cancer and has been defined as the characterization and measurement of biological processes in living animals and human patients at the cellular and molecular level. To achieve truly targeted imaging of specific molecules which occur in relatively low concentrations in living tissues, imaging techniques must be highly sensitive. Although US, CT and MRI are often considered to be molecular imaging modalities, in practice, scintigraphic and optical imaging are the most relevant to the above definition and are used most frequently because of their true targeted detection capabilities. Table 1 compares some of the advantages and disadvantages of the different imaging modalities for *in vivo* evaluation of molecular processes. Each modality has unique strengths in terms of sensitivity, spatial resolution, temporal resolution, cost, and depth of tissue penetration.

**Table 1:** Various modalities used in molecular imaging.

Modality	Advantages	Disadvantages
Optical imaging	Non-ionizing radiation. High sensitivity for ligand detection. Sub-cellular spatial resolution. Allows for visualization of subtle anatomic abnormalities. Can be quantitative High target to background ratios. Reasonable doses can be used repeatedly without harm to the patient.	Poor or limited tissue depth penetration makes imaging of some body parts inaccessible.
Scintigraphic	Very high sensitivity for ligand detection. Hundreds of agents already tested in the clinic. Many agents approved for human use. Potential for whole body scanning.	Radiation dose decreases utility in low-risk screening applications. Poor spatial and temporal resolution. Radioactive compounds (Radiotracers) used which have an intrinsically limited half-life and expose the patient and practitioner to ionizing radiation. Subject to stringent safety regulations which limits repeated use.
Magnetic resonance imaging	High spatial resolution and simultaneous intrinsic anatomic correlation.	Low sensitivity for ligand detection.
Ultrasound	Inexpensive. Images are generated in real time with high temporal resolution.	Very difficult to image extravascular molecular targets.

Recently, researchers have developed multi-modal imaging strategies that combine optical, MR and nuclear processes to enhance validation. These integrated techniques aim to confirm observed biological phenomena using independent views and to better delineate the localization and expression of molecular biomarkers. Moreover, the combination of imaging methods and probes work synergistically to improve sensitivity for the investigation of biological processes. In this review, we describe the most common molecular imaging modalities: scintigraphic, optical, MRI, US, and multi-modal imaging and present the current status of molecular probes.

Each imaging modality presented in Table 1 uses exogenously administered imaging agents that use a unique mechanism to generate images with high contrast and molecular specificity. Small molecules, peptides, antibodies, aptamers, nanoparticles, and quantum dots are classes of molecular probes that have been extensively used in cancer imaging research. Table 2 provides an overview of the general classes of exogenous targeting agents that are frequently employed across all imaging modalities. Progress in this field is becoming more advanced due to multi-disciplinary collaborations among chemists, molecular biologists, clinicians, physicists, and imaging scientists. Today, the molecular imaging community is rapidly advancing the performance of imaging instruments and the capability of molecular probes.

**Table 2:** Various ligands for imaging in different molecular imaging modalities.

Detection ligand	Advantages	Disadvantages
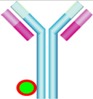	Low antigenicity and acceptable toxicity and high specificity to targets. Many clinically approved antibodies available for labeling.	Long blood half-life decreases specificity of signal.
(Antibody)		
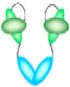	These structures retain high binding affinity and specificity. Clearance times well-suited for imaging.	More complex to formulate compared to whole antibodies.
(Antibody fragments)		
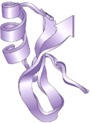	High specificity to targets, easy for synthesis and feasible for conjugation with contrast agents, rapid clearance times.	Many peptides have brief serum half-lives, usually caused by degradation or excretion.
(Peptides)		
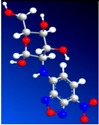	High specificity, rapid clearance. Intra-cellular targets are available for imaging.	Fluorochromes and their comparable size to small molecules may affect pharmacokinetics and biodistribution of the resulting labeled ligands.
(Small molecules)		
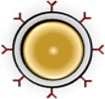	Can be used for multi-valent targeting, strong fluorescence, broad and narrow excitation bands, tunable fluorescence, and ideal for efficient modification due to large surface area.	Long term toxic effects generated by tracers of heavy metals, requires long clearance times.
(Nanoparticles)		

## 2. Scope of the Review

Recent research findings have demonstrated the development and application of various imaging agents in different modalities. This includes bioluminescent reporters, activatable probes, nanoparticles, quantum dots, radionuclide probes, metal chelate probes, and microbubbles [7,8,9,10,11,12,13,14,15,16,17,18,19,20,21,22,23,24,25]. This review will focus on the current status and use of these targeted molecular probes, including dual labeled probes. Moreover, we will discuss recent developments in multi-modal probes associated with nanoparticles, MRI agents, optical contrast agents and radionuclides and how these probes can be used synergistically in multi-modal imaging.

## 3. Imaging Modalities

Here, we discuss non-invasive, *in vivo* imaging technologies, including scintigraphic, optical, MRI, and ultrasound, which are rapidly evolving in the expanding field of molecular imaging.

### 3.1. Optical Imaging

**Figure 1:**
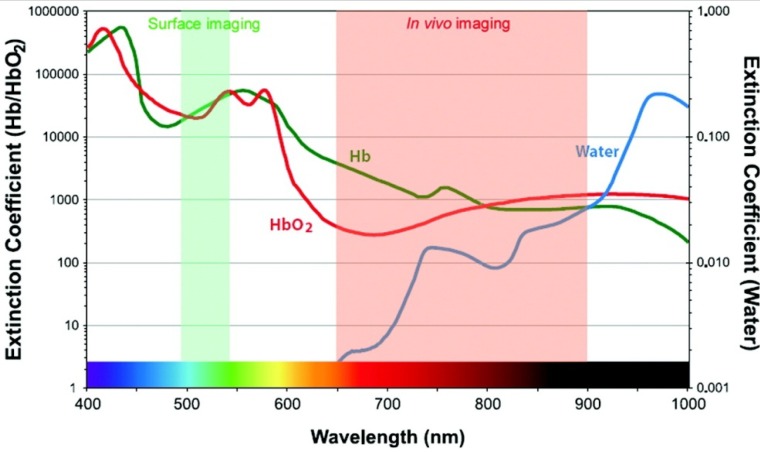
Hemoglobin and water in tissue absorb light over a broad spectral regime, creating an optical window in the NIR spectral band between 650 and 900 nm that represent an optimal tradeoff between image resolution and tissue penetration for *in vivo* imaging. (Reproduced from [17] with permission)

Optical imaging uses light from the visible and NIR regimes, and has key advantages for real time performance and sub-cellular resolution. The detectors used are sensitive to a broad range of wavelengths, and can be image multiple probes in different bandwidths to perform multi-spectral imaging. As compared to whole body imaging systems, these instruments are, in general, more portable and less expensive. Optical imaging techniques with contrast agents are commonly used for *in vitro* and *ex vivo* applications in molecular and cellular biology (e.g., fluorescence microscopy). Blue and or green contrast agents achieve higher spatial resolution at the expense of less tissue penetration. However, these agents are also sensitive to background tissue autofluorescence and hemoglobin absorption, as shown in Figure 1. Thus, an optical window in the 665 to 900 nm range results in an optimal tradeoff between image resolution and penetration depth for *in vivo* imaging. Several NIR fluorescent dyes have become available recently that are either coupled to affinity ligands (peptides, antibodies) or that are activatable [26]. The most common optical imaging techniques include bioluminescence, confocal microscopy, two-photon microscopy, fluorescence endoscopy, and fluorescence molecular tomography. The working principles of these instruments are discussed in detail in the references [27].

### 3.2. Scintigraphic Imaging

Scintigraphy is a common mode of imaging used in nuclear medicine. Radioisotopes are administered intravenously, and the emitted radiation is captured by external detectors (gamma cameras) to form two-dimensional (planar) images. PET and SPECT use this technique to form three-dimensional images. Applications of scintigraphy include tracking drug biodistribution, monitoring therapeutic response, and evaluating the physiological response. This imaging modality has a key advantage that only trace amounts of the imaging agent are needed for whole body detection, ranging from subpicomolar to picomolar. These tiny amounts do not affect either the biodistribution of the probe or physiological processes involved in its distribution. However, scattering of radiation as it passes through the body does result in reduced image resolution. PET/SPECT was first demonstrated in the 1970s as clinical research tools. These methods have matured over this period of time and became ready for clinical use. In both techniques, an imaging agent is created by labeling an affinity ligand with a radioisotope. PET utilizes positron-emitting radioisotopes created in a cyclotron, including ^18^F, ^11^C, ^64^Cu, ^124^I, ^86^Y, ^15^O and ^13^N, or in a generator, including ^68^Ga, and detects emitted γ rays. The most widely used isotope is ^18^F because of practical reasons associated with a half-life of 109.8 min. At present, substrates labeled with ^18^F, including FDG for hexokinase in glucose metabolism and NaF for bone imaging, are FDA approved PET tracers. SPECT detects γ emitters such as ^123^I, ^111^In, ^99m^Tc, ^67^Ga, and ^201^Tl. These imaging agents are readily available and are relatively less expensive than PET tracers.

PET and SPECT have strengths that complement each other. In general, PET offers superior resolution, compared to SPECT, and the resolution is intrinsically limited to the distance traveled by the positron before it becomes annihilated (up to several millimeters, depending on the positron energy) [28]. Another advantage of PET, compared to SPECT, is superior quantification due to its relatively higher energy (511 KeV). However, improvements in SPECT technology are closing these gaps [29,30]. Advantages of SPECT over PET include lower cost, broader choice of FDA approved radionuclides, and flexibility to distinguish multiple emission energies simultaneously. The last feature allows SPECT to distinguish co-administered tracers that differ in their targets and emission energies [31]. Moreover, the use of ionizing radiation restricts the number of research studies that can be performed in a single patient. As a result, the dose is kept low (100 to 1000 Megabecquerel MBq), and is further limited if the tracer’s biodistribution results in accumulation in a particular organ(s).

In summary, although PET and SPECT are different imaging technologies, practical differences in the physical interpretation of images are subtle. The major differences between these two imaging modalities lie in the chemistry of the radiopharmaceuticals (*i.e.,* design and synthesis), which in turn arises from different chemical attributes of the accessible positron and gamma emitting radionuclides. The most important positron emitter is ^18^F, a small atom that is well suited for covalent bonding to small organic molecules, such as metabolites and amino acids, and can retain the chemical structure of the parent compound without drastic alterations to their structure and function. As discussed previously, the short half-life of ^18^F results in rapid pharmacokinetics, and chemical synthesis of these radiolabeled molecules must be coordinated with the time and place of use. Moreover, the synthesis process is complex and the radiochemical facilities are costly to set up. While metal based radionuclides are important in SPECT, their use is limited because they require binding to complex chelator molecules and cannot be incorporated into small molecules without interfering with functionality. This requirement results in a significant increase in the size of the small molecule used. Thus, metals are better suited for larger molecules, such as peptides, polymers, antibodies, and proteins, where structural changes that result from labeling incur less disruption to original functionality of the affinity ligand. The longer half-life of SPECT radionuclides is also generally better suited for the slower pharmacokinetics of these larger molecules. 

### 3.3. Magnetic Resonance Imaging (MRI)

MRI has become increasingly more popular for both research and clinical imaging because of its non-invasiveness and capability for producing three dimensional images with high spatial and temporal resolution. Approximately 35% of all clinical MRI scans utilize contrast media. However a primary limitation of this imaging modality is the sensitivity of contrast agents and the requirement for high concentrations that vary from 0.1 to 0.6 mM [32]. Typically, MR images visualize anatomical structures based on their water content by measuring signals generated from protons in response to excitation by radio-waves that match the intrinsic frequency of precessing protons. In molecular imaging, the strength of the MR field is tailored for the detection of specific cells or even molecules inside microanatomical structures at high resolution. Metals with magnetic moments (Gd^3+^, Mn^2+^, and Fe^3+^) are effective contrast agents for the MRI, discussed later in section 6.

### 3.4. Ultrasound

Ultrasound is one of the most common imaging methods used to evaluate tumors in the thyroid, breast, prostate, liver, pancreatic, ovarian, uterine and kidney, and is frequently used to guide biopsies. US generates images in real time (high temporal resolution), and is used for functional imaging. Another advantage of this technique is that there is no ionizing radiation and serial follow up studies can be performed over time. US contrast agents used in molecular imaging include encapsulated microbubbles, liposomes, and perfluorocarbon emulsions [33,34]. US generates images by detecting differential reflections of sound waves, and signal enhancement occurs when these agents oscillate in response to acoustic pulses, making them more reflective than normal tissue. Because of the small size of these agents (0.1 to 8 μm in diameter) they are restricted to the intravascular space; therefore, molecular imaging applications have been limited to vascular processes, such as angiogenesis, inflammation, and thrombosis. Notably, ongoing efforts are under way to design hybrid probes that can carry therapeutic agents, providing simultaneous targeted delivery of a contrast agent and a drug. In general, US is less sensitive than scintigraphic imaging, and has lower spatial resolution than MR imaging. 

### 3.5. Multi-Modal Imaging Platform

Because each imaging modality has its unique advantages, combining imaging modalities may provide better results and cross-validate findings. The combined use of PET and CT is an example of a successful multi-modal imaging platform where CT provides high-resolution anatomical details that can be used to register functional data provided by PET [35]. Currently, there are very few imaging probes that can be detected by more than one modality; however, dual agents for radionuclide and optical [36,37] or MR and optical [38,39,40] have been demonstrated. Another multi-spectral approach in optical imaging that has been successfully applied *in vivo* involves applying two or more optical agents that are distinguished by their fluorescence emission [41,42,43]. For example, confocal microscopy has been combined with conventional white light endoscopy, and autofluorescence bronchoscopy has been integrated with OCT (Optical Coherence Tomography) [44,45]. We expect that other combinations of high-resolution and wide-field optical imaging methods to follow. Multi-modality systems may provide a number of distinct advantages, including improving the ability to quantify and locate molecular processes and characterizing new imaging probes [46]. However, to date, technical and practical issues have made it challenging to combine and translate these techniques into the clinic.

## 4. Molecular Probes for Optical Imaging

Targeted imaging with optical probes consists of three main components: (1) signaling moiety for detection; (2) carrier molecule for optimized pharmacokinetics, and (3) affinity ligand for target binding. Small molecules have better pharmacokinetics and faster clearance than large molecules. Other important criteria for designing an effective probe include: (1) endogenous properties of the tissue that generate contrast; (2) selection of fluorescent dyes; (3) size and type of linker, and (4) features of the affinity ligand. Thus, the development of the probes has always been one of the central focuses of molecular imaging. To obtain an accurate diagnosis of disease in an early stage and to evaluate the response to therapy, several strategies have been employed for developing novel optical imaging probes. A clinically useful probe provides a high “target-to background” ratio to maximize the *in vivo* image contrast. The ideal imaging compound has high binding affinity, specific uptake and retention in the target, rapid clearance from non-target tissues, high stability and integrity *in vivo*, ease of preparation, and safe clinical use. This section reviews the key components of exogenous molecular probes including, organic fluorescence dyes, peptides, antibodies, small molecules, and summarizes the different targeting principles associated with the design of these probes.

### 4.1.Non-specific Optical Contrast Agents

**Figure 2:**
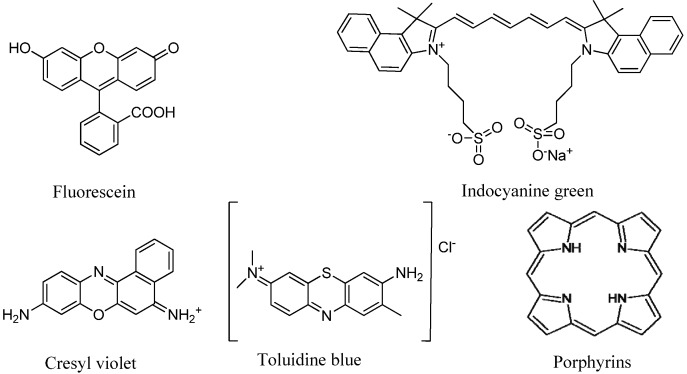
Chemical structures of common non-specific optical contrast agents used in clinical research.

The first agents used in optical imaging include non-specific small molecules that have distinct absorbance maxima, unique fluorescence properties, or inducible changes in the presence of diseased tissue. These non-specific contrast agents enhance visualization of changes in cell morphology that occur during cancer transformation. A range of dyes that have distinct optical properties include fluorescein, indocyanine green, cresyl violet, toluidine blue and Lugol’s iodine. In addition, porphyrins, chlorins, and rare earth metal chelates of terbium and europium have been used, as shown in Figure 2. These non-specific dyes are currently being used in the clinic for research purposes, and can produce intra- or extra-cellular localization based on their size or charge distribution. Because of their low molecular weights (≤1 kD), these small molecules can be delivered efficiently via either topical or intravenous routes. However, their use can be limited by relatively high levels of non-specific interactions that increase background. Several studies with indocyanine (ICG) dyes have been conducted [47]. In such a recent study, Kusano *et al.* found high false positives (~30%) with injection of 2 mL of ICG dye in the subserosa around the tumor, resulting in unnecessary follow-up procedures [48]. 

### 4.2. Target Specific Molecular Probes

Image contrast and diagnostic accuracy can be improved significantly by using targeted contrast agents to enhance the signal from cancer specific molecules. Regardless of the imaging agent employed, targeting of disease specific markers is a much more powerful means for providing diagnostic information and can also be used to guide therapy. Fluorescence dyes can be conjugated to specific substrates to form a molecular probe that visualizes specific tumor targets. Key factors to be considered for selecting the targeting platform include agent bioavailability, feasibility of synthesis, low toxicity, specific target uptake and retention, rapid clearance from non-target tissues, high target binding affinity, and high signal-to-noise ratio. Various conjugates have been reported that satisfy most of the above properties and will be discussed below.

#### 4.2.1. Conjugates with Peptide Ligands

After the discovery of several specific peptide receptors in various forms of cancer, peptide analogs were introduced for cancer imaging because of their good stability, receptor binding properties, and pharmacokinetic behavior. Applications include the detection and staging of neuroendocrine tumors, adenocarcinomas, lymphomas, and melanomas. Various optical contrast agents, such as visible and NIR dyes, have been used for labeling. However, recent efforts have focused on cyanine based NIR dyes for optical imaging because they provide deeper tissue penetration [49,50,51,52]. In this section, we will focus on peptide-based fluorescence probes and their use for targeted imaging in cancer.

Peptides can be labeled with a variety of fluorophores that have different excitation and emission wavelengths. Fluorophores in the visible range, include 7-amino-4-methylcoumarin (AMC), fluorescein isothiocyanate (FITC), fluorescein carboxylic acid, and 5-carboxytetramethylrhodamine (TAMRA). Tissue has significantly less autofluorescence at longer wavelengths (650–900 nm) [53]. Chemical structures and optical properties of most NIR dyes are similar to that of indocyanine green, an FDA-approved indotricarbocyanine dye commonly used in angiography. To date, a number of NIR dyes have been reported, and their reactive intermediates for peptide bioconjugation are commercially available. Examples include the family of Cy dyes from GE Healthcare, Alexa Fluor dyes from Invitrogen, IRdye dyes from Li-COR Bioscience, SRfluor dyes from Molecular Targeting Technologies, HyLyte fluor from Anaspec, and CF633 from Biotium. Many biologically active peptide analogs reported in the last decade have been labeled with NIR and visible fluorophores.

**Figure 3:**
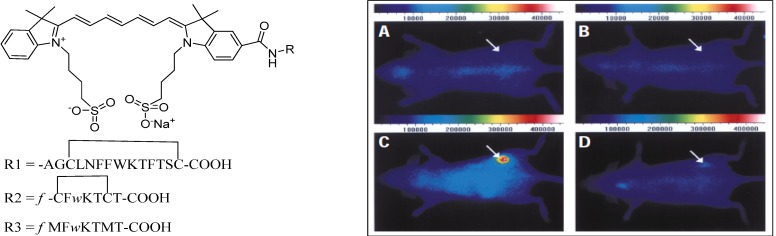
(Left panel) Chemical structure of ITCC conjugated somatostatin (R1), octreotate (R2) and M2M7 (R3). (Right panel) *In vivo* fluorescence images of RIN38/SSTR2 tumor-bearing nude mice before intravenous injection of (A) R2 and (B) R3. Fluorescence images acquired 6 h after intravenous injection of (C) R2 and (D) R3 at a dose of 0.02 μmol/kg body weight are shown. (Reprinted with the permission from [54].)

**Figure 4:**
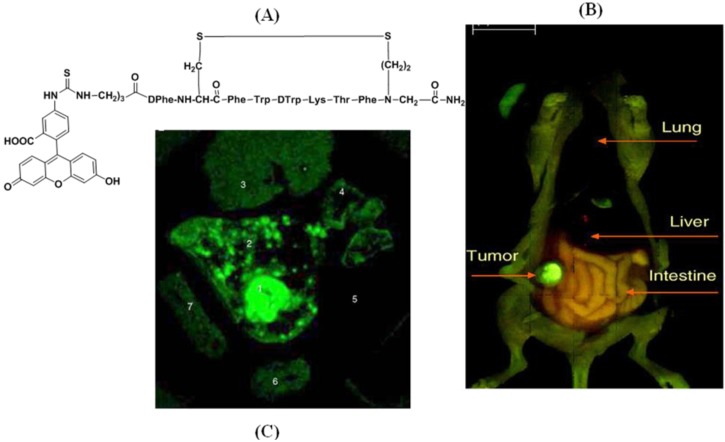
(A) FITC conjugated peptide probe. (B) Whole body fluorescence image of a mouse whose abdomen has been surgically exposed, revealing a subcutaneous H69 tumor at 24 h after the administration of the FITC-labeled peptide. (C) Fluorescence image of an excised tumor and adjacent normal organs: (1) H69 tumor nodule; (2) skin; (3) liver; (4) lung; (5) pancreas; (6) kidney; and (7) spleen at 24 h post-administration of the FITC-conjugate peptide. (Reprinted with permission from [55].)

The *in vivo* diagnostic use of a NIR-dye conjugate consisting of indotricarbocyanie (ITCC) dye and octreotate for tumor imaging has been reported, as shown in Figure 3. The ITCC-octreotate conjugate exhibits fast receptor binding properties in SSTr-2-overexpressing RIN38/SSTr-2 cells and provides three-fold higher tumor fluorescence intensity in mice bearing RIN38/SSTr-2 tumors from 3 to 24 h after intravenous injection [54]. Different somatostatin analogs have also been labeled with fluorescein and rhodamine dyes and tested in SSTr-positive NCI-H69 human small cell lung cancer (SCLC) tumors and HT-29 colon tumor-bearing mice, respectively [55,56]. This result demonstrates for the first time that human SCLC can be specifically targeted with high selectivity by a fluorescent bioconjugate of a somatostatin analog, providing high target-to-background (9–90) ratios, as shown in Figure 4. 

Examples of molecular probes based on a peptide platform include bombesin conjugated to Alexa Fluor 680 (7–14) via a Gly-Gly-Gly linker. This probe has been synthesized for targeted gastrin-releasing peptide receptor (GRPr) imaging and demonstrated specific GRPr targeting ability *in vitro* and *in vivo* in mice bearing T-47D breast cancer cells [57]. In addition, an enormous number of optical probes associated with angiogenesis-specific targets have been developed utilizing RGD peptide analogs. Chen *et al*. developed a Cy5.5 conjugated c(RGDyK) via an amide bond to the ε-amino group of the lysine residue and used this probe to image integrin α_v_β_3_-positive U87MG tumor xenografts in mice [58]. In a separate study conducted by Cheng and Wu *et al*., Cy5.5- or Cy7- conjugated mono-, di-, and tetrameric RGD peptides were developed, and demonstrated increased receptor binding affinity compared to that of their monomeric counterpart [59,60]. 

Furthermore, phage display, an unbiased, high-throughput method developed from recombinant DNA technology, has been used to generate a library of clones that bind preferentially to cell surface targets [61]. Peptides have been selected using this technique against a secreted protein acidic and rich in cystein (SPARC) for detecting invasive cancer [62] and against vascular cell adhesion molecule-1 (VCAM-1) for identifying inflammatory endothelium [63]. Dye-labeled phage clones exhibited excellent *in vivo* targeting ability in tumors and in VCAM-1 expressing vessels, suggesting that fluorophore-based phage clones can be used as *in vivo* imaging probes.

**Figure 5:**
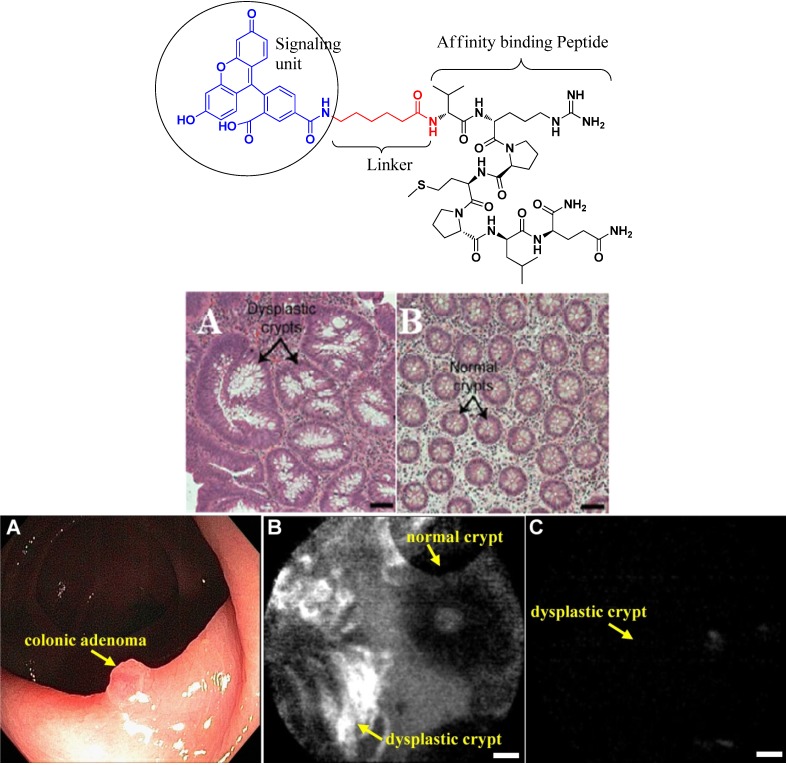
(Top panel) Chemical structure of phage derived peptide with sequence VRPMPLQ conjugated with fluorescein via an aminohexanoic linker. (Middle Panel) Histology of colonic (dysplasia) adenoma (A) and normal mucosa (B) stained with H&E. Scale bars, 50 μm. (Bottom panel) *In vivo* validation of peptide binding. (A) Conventional white light endoscopic image of colonic adenoma. (B) *In vivo* confocal image following topical administration of peptide probe, demonstrating preferential binding to dysplastic crypt (left half) and no binding to normal crypt (right half). (C) Confocal image with control peptide (scrambled sequence QLMRPPV) shows no binding to dysplastic crypt, scale bar 20 μm. (Reprinted from [64] with permission.)

Similarly, we have successfully used this technique to identify peptides that bind preferentially to dysplastic rather than to normal colonic mucosa, and performed imaging with a confocal fluorescence microendoscope to validate preferential binding *in vivo* [64]. The human colonic dysplasia-specific peptide VRPMPLQ was conjugated to fluorescein via an amino-hexanoic acid linker, as shown in Figure 5. When an adenoma was observed on white light colonoscopy, approximately 3 mL of the fluorescence-labeled peptide VRPMPLQ diluted in PBS at a concentration of 100 μM was administered topically to the adenoma and surrounding normal mucosa. After 10 minutes for incubation, the unbound peptide was gently rinsed off, and the flexible fiber confocal microendoscope was passed through the instrument channel, and placed into contact with the mucosa for collection of fluorescence images.

**Figure 6:**
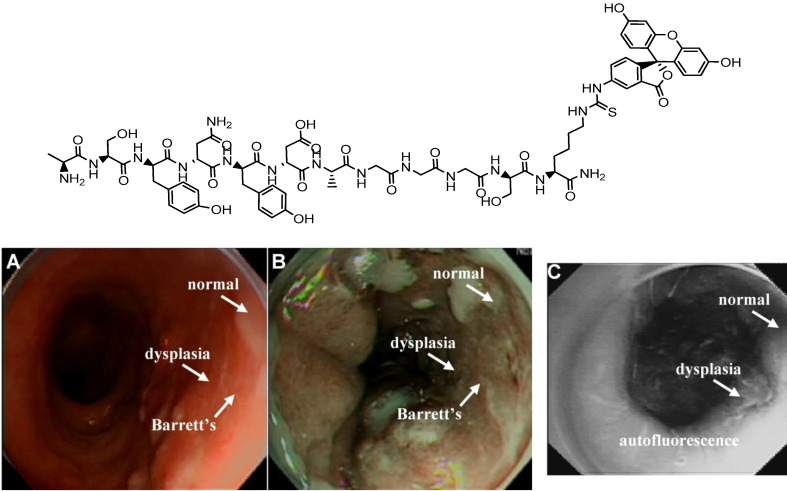
(Top panel) Chemical structure of the candidate peptide ASYNYDA selected using techniques of phage display to target high-grade dysplasia (HGD) in Barret’s esophagus. (Bottom panel) *In vivo* localization of peptide binding to HGD on wide-area fluorescence endoscopy. (A) Conventional white light image of the distal esophagus shows endoscopic evidence of intestinal metaplasia but no sign of pre-malignant lesions (dysplasia). (B) Narrow band image (NBI) of the same region shows improved spectral contrast, highlighting intestinal metaplasia but not dysplasia. (C) *In vivo* fluorescence image following topical administration of affinity peptide reveals preferential binding to HGD. (Reprinted from [20] with permission.)

Using a similar selection strategy for pre-malignant esophageal mucosa, phage with the peptide sequence ASYNYDA was identified. The candidate peptide was synthesized by incorporating Gly-Gly-Gly-Ser linker which is used in generating the phage library to expose the peptides on PIII protein of phage virion. A lysine residue was incorporated at the C-terminus and side chain of this lysine was used for labeling FITC. The use of topically applied fluorescence-labeled peptides to target high-grade dysplasia on wide-area endoscopy has been demonstrated *in vivo* in Barrett’s esophagus, as shown in Figure 6. Figure 6A shows a conventional white light image of Barrett’s mucosa in the distal esophagus *in vivo*; no distinct architectural lesions associated with neoplasia can be seen. Figure 6B shows the corresponding narrow band (NBI) image of the same region of the esophagus, and highlights the presence of intestinal metaplasia, distinguished by the brown color, but this non-specific contrast enhanced image is not sensitive to the presence of dysplasia. In Figure 6C, the fluorescence image collected following topical administration and incubation of the peptide reveals a region of high-grade dysplasia which was further confirmed with guided biopsy collected from the peptide bound region and confirmed by gastrointestinal pathologists.

Thus, fluorophore-conjugated peptides represent a powerful tool for targeted imaging in the clinic as well as in pre-clinical settings.

#### 4.2.2. Conjugates with Antibody Ligands

Targeted imaging using so called immunoconjugates has also been demonstrated with antibodies and their modified analogs, including antibody fragments, diabodies and minibodies. The strategy for coupling cyanine dyes as labels to affinity-maturated single-chain antibody fragments directed against an angiogenesis-specific oncofetal isoform of fibronectin (ED-B fibronectin) for *in vivo* imaging was demonstrated by Folli *et al*. [65] and Ballou *et al*. [66]. When Cy7 or Cy5.5-labeled antibody fragments were injected, the expression of ED-B-fibronectin was visualized in tumour-bearing mice, a mouse atherosclerosis model, and angiogenesis induced in the cornea of rabbits, demonstrating the relevance of ED-B-fibronectin as a marker of angiogenesis [67,68,69]. In separate studies conducted by various independent research groups, cyanine dyes conjugated to antibody- and protein-based ligands with epidermal growth factor EGF [70], endostatin [71], and endothelial-expressed glycoproteins were used for optical imaging of inflammation and lymph nodes [72].

ICG dye has been approved by the FDA for intravenous use to measure cardiac output, assess hepatic function, and visualize ocular vessels. This dye has been conjugated with antibodies for targeted detection of cancer in the digestive tract. Reactive derivatives of ICG including ICG-*N*-hydroxysulfosuccinimide ester (ICG-sulfo-OSu) and 3-ICG-acyl-1,3-thiazolidine-2-thione (ICG-ATT) have been developed and labeled with anti-CEA and anti-MUC1. These conjugated compounds exhibit small shifts in the excitation and emission wavelengths relative to pure ICG, and have been used to demonstrate the proof of principle for targeted endoscopic imaging [73]. Furthermore, monoclonal ICG-labeled mouse anti-CEA antibody has been used to target gastric cancer on biopsy specimens and imaged with NIR endoscopy. A conventional white light endoscopic image was collected first from freshly resected gastric mucosa, and then a NIR fluorescence image of the same specimen stained with ICG labeled anti-CEA antibody was acquired, revealing foci of cancer. In addition, specimens of normal gastric mucosa did not reveal antibody binding, and were used as controls. 

**Figure 7:**
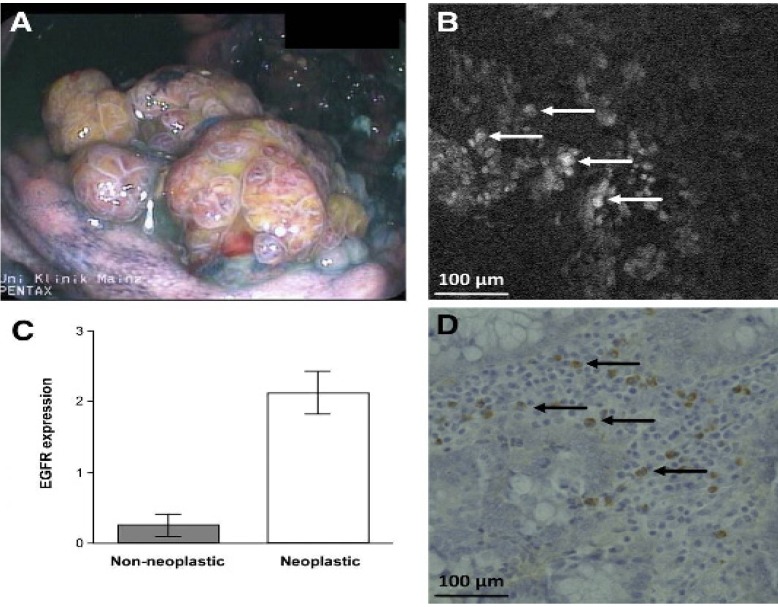
Targeted imaging with fluorescent-labeled anti-EGFR antibody. (A) White light image of colonic neoplasia on colonoscopy. (B) Confocal microendoscopy image reveals increased fluorescence intensity within the lesion (arrows), scale bar 100 μm. (C) Mean fluorescence intensity on human specimens was 0.25 ± 0.16 for normal mucosa *vs.* 2.12 ± 0.30 for neoplasia (p < 0.002). (D) Immuohistochemistry demonstrates EGFR expression in multiple tumor cells (arrows). (Reprinted with the permission from [74].)

In a recent study, Goetz *et al*. used a fluorescent-labeled whole antibody to target epidermal growth factor receptor (EGFR), and demonstrated binding of this antibody conjugate against cells in culture with either low or high EGFR expression. This probe was also validated on xenografts grown subcutaneously in mice and on *ex vivo* human samples. Imaging was performed using a confocal microendoscope with 488 nm excitation, and high-resolution fluorescence images were collected from 505 nm to 585 nm. This study demonstrates that molecular imaging is feasible by targeting EGFR, as shown in Figure 7 [74].

#### 4.2.3. Conjugates with Small Molecule Ligands

**Figure 8:**
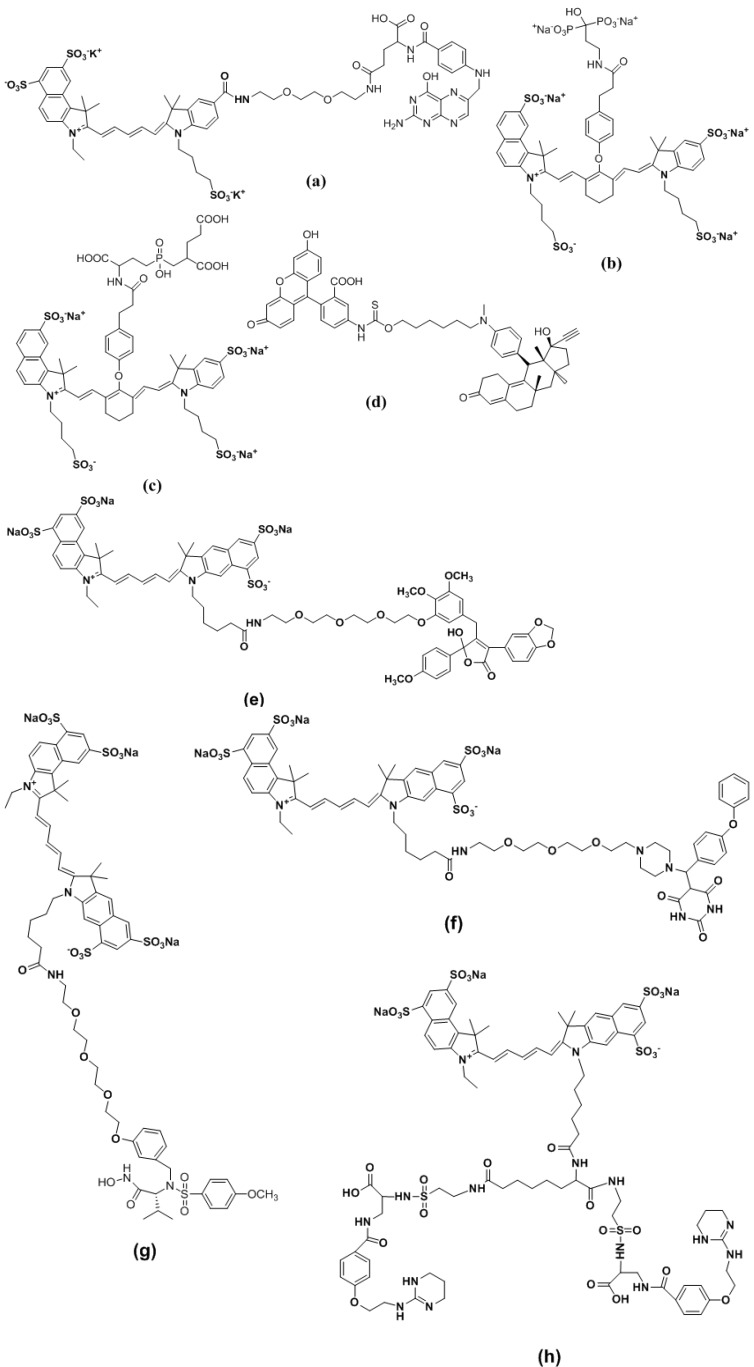
Chemical structures of fluorophore conjugates with small molecule ligands; (a) Cy5.5 dye conjugated with folic acid via a PEG linker; (b) bone-targeted IRDye78 pamidronate Pam78; (c) IRDye78 conjugate with PSMA (Prostate Specific Membrane Antigen) ligand GPI; (d) progesterone receptor antagonist mifepristone labeled with FITC; (e) Cy 5.5 dye conjugated photoprobe for ETAR receptor; (f) Barbiturate based Cy5.5 conjugated probe for monitoring MMPs; (g) Hydroxamic based Cy5.5 conjugated probe for MMPs and (h) computationally screened Cy5.5 labeled probe for α_v_β_3_ integrins.

**Figure 9:**
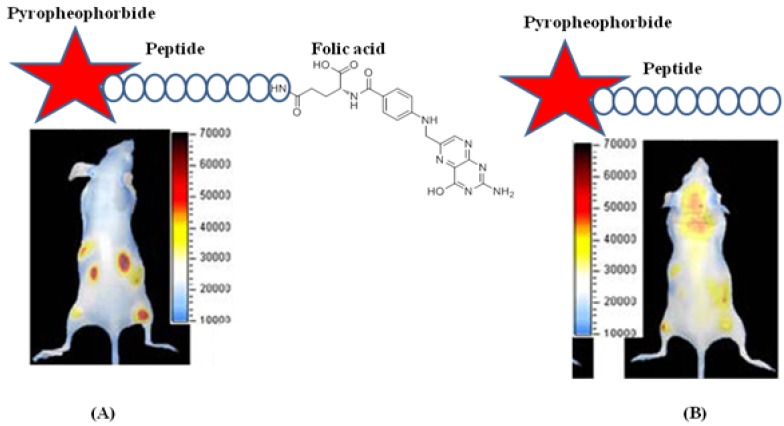
Bioluminescence images of mice with double xenograft tumors, HT 1080 (left) and KB (right), collected 6.5 h after injection of (A) Pyro-Peptide-Folate (PPF), revealing targeting of the folate receptor positive KB tumor and of (B) Pyro-Peptide (PP) without folic acid, demonstrating no signal for either HT1080 or KB tumor. (Reprinted from [78] with permission.)

Unlike the non-specific dyes discussed in the section 4.1, small molecules can be effective contrast agents for targeted imaging because of their relative ease of delivery into tissue, ability to mediate specific molecular interactions, and rapid uptake by tumor cells. Several small molecule affinity ligands conjugated with fluorescence dyes are shown in Figure 8. Among these, folic acid, cobalmin, 2-[*N*-(7-nitrobenz-2-oxa-1,3-diazol-4-yl)amino]-2-deoxy-d-glucose (NBDG, a fluorescent analog of FDG), and bis-phosphonate pamidronate/alendronate are frequently used for targeted imaging. Folic acid is a promising ligand that has been employed as carrier molecule to target the abundant folate receptors on proliferating cells and activated macrophages. After conjugation of folic acid with the cyanine dye Cy5.5 or NIR2, uptake studies demonstrated promising imaging properties in cancer models [75,76] similar to that of peptide ligands and showed utility for the early detection of inflammatory disease in a rheumatoid arthritis model [77]. Stefflova *et al.* developed the PDT agent pyropheophorbide conjugated with a small peptide linker and folic acid for the folate mediated targeting probe which improves the efficiency of targeted delivery of NIR imaging and PDT agents, as shown in Figure 9 [78]. In a separate work, Holtke *et al*. successfully developed and validated a novel fluorescent photoprobe for imaging endothelin A receptors (ETAR). Starting from the high-affinity selective ETAR antagonist 3-benzo[1,3]dioxol-5-yl-5-hydroxy-5-(4-methoxyphenyl)-4-(3,4,5-trimethoxybenzyl)-5H-furan-2-one (PD 156707), this group developed the Cy5.5 conjugated ETAR selective photoprobe by modifying the lead structure with a PEG-spacer containing an amino moiety for facilitating labeling with Cy5.5 ([79], Figure 9e). Further evaluation of this photoprobe was conducted using human carcinoma cell lines with different degrees of ETAR expression. Fluorescence microscopy revealed that ETAR-positive MCF-7 human breast adenocarcinoma and HT-1080 human fibrosarcoma cells effectively bind the photoprobe at very low doses (nM), while ETAR-negative MDA-MB-435 human breast cancer cells showed no fluorescence signal. Faust *et al.* presented a pyrimidine-2,4,6-trione (barbiturate) based Cy5.5 conjugated non-peptidyl lead structure for developing an imaging agent to visualize activated matrix metalloproteinases (MMPs) *in vivo*. Proof of principle was confirmed by treating the candidate probe with a high (A-673) and moderate (HT-1080) MMP-2 secreting cell line by fluorescence microscopy, which showed efficient binding of the probe to MMP-2 positive cells while the optical model tracer did not bind to the MMP-2 negative cells (MCF-7) ([80], Figure 9f). In another report [82], a hydroxamic acid based Cy5.5 conjugated probe was developed for MMPs and tested against the low and high expression of MMP in cell lines ([81], Figure 9g). Very recently, Li *et al*. presented a computer model to identify new inhibitors for specific binding with α_v_β_3_ integrin receptors. A near-infrared (NIR) fluorescent dye Cy5.5 was labeled with this computationally screened probe and tested against U87 cells that over express α_v_β_3_ and further applied *in vivo* in U87 mouse xenografts ([82], Figure 9h).

### 4.3. Activatable Probes

**Figure 10:**
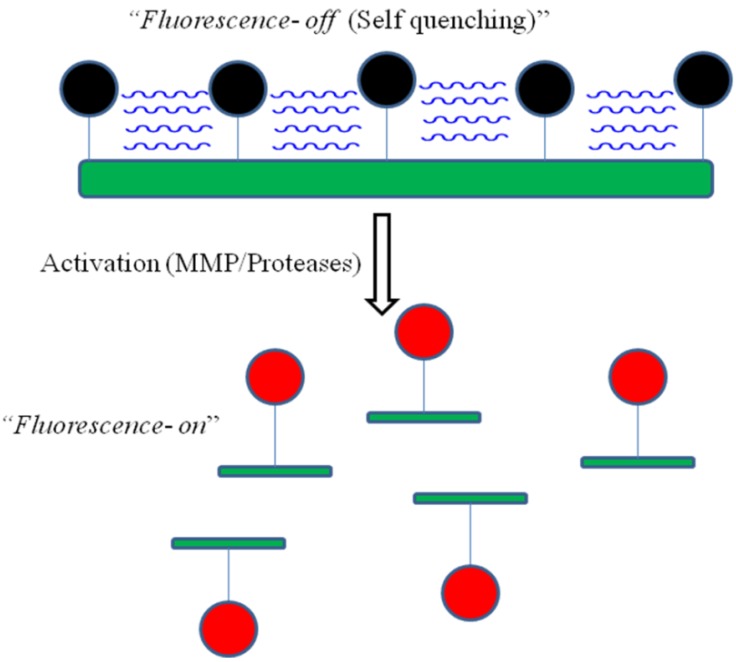
Schematic diagram of enzyme activated methoxypolyethylene modified *poly-L-lysine* probe. In the dormant state, the proximity of the fluorochrome molecules to each other results in fluorescence quenching. The presence of over expressed enzymes (e.g., MMP or protease) cleaves the probes, releasing intense fluorescence. (Modified and reprinted from [83] with permission).

Activatable or “smart” probes are another class of molecular imaging agent that releases fluorescence in the presence of factors present only in the diseased tissue microenvironment. Fluorescence is released when a fluorophore is converted from a quenched, non-emitting state into a free state. Compared to the fluorophore-labeled peptides, an activatable probe is optically “dark” in its quenched state and becomes strongly fluorescent *in vivo* following enzymatic cleavage. This idea was initially developed by Weissleder *et al*. by conjugation of the Cy5.5 dye to the backbone of the poly-l-lysine (PL) which was sterically protected by multiple methoxypolyethylene glycol (MPEG) side chains as a delivery vehicle for quenched fluorochromes to tumors [83]. Each PL backbone contained an average of 92 MPEG molecules and 11 molecules of Cy5.5 yielding (Cy5.5)11-PL-MPEG92 (abbreviated as C-PGC) as the candidate targeting probe. Conjugation of the fluorophores either directly or via cleavable peptide sequences in close proximity to each other causes strong fluorescence quenching. As schematically illustrated in Figure 10, enzymatic cleavage by cathepsins, MMPs and other proteolytic enzymes activates the probe by liberating single dye-loaded fragments either through backbone cleavage of the lysine-lysine amide bonds or cleavage at the peptide sequences. 

A wide range of applications has been demonstrated with enzymatic degradation in many disease models, including cancer, atherosclerosis, rheumatoid arthritis, and thrombosis using this simple principle. Self quenching is generally not as efficient as that of custom designed molecular quenchers, such as QSY7 and dabcyl group. Ogawa *et al*. demonstrated a new class of activation mechanism using the TAMRA (fluorophore)-QSY7 (quencher) pair. This design is based on the conjugation of this fluorophore-quencher pair to either avidin (targeting the D-galactose receptor) or to trastuzumab (an FDA approved monoclonal antibody against the human epithelial growth factor receptor type2 (HER2/neu)). Its performance in mouse models of cancer has been demonstrated. Two probes, TAMRA-QSY7 conjugated avidin (Av-TM-Q7) and trastuzumab (Traz-TM-Q7) have been synthesized. Both demonstrated better performance than that of similar self-quenching probes. *In vitro* fluorescence microscopy studies of SHIN3 and NIH/3T3/HER2+ cells demonstrated that Av-TM-Q7 and Traz-TM-Q7 produced high intra-cellular fluorescence compared to the negative control conjugate Dac-Tm-Q7. *In vivo* imaging with Av-TM-Q7 and Traz-TM-Q7 in mice has can detect small tumors, demonstrating proof of principle for this design [84].

Recently, Urano *et al*. developed a novel approach for activatable probes that utilizes the photo-induced electron transfer (PeT) mechanism [85]. At physiologic pH (~7.4), a non-protonated *N*,*N*-dialkylated aniline moiety is able to virtually eliminate fluorescence emission from the independent 2,6-dicarboxyethyl-1,3,5,6-tetramethyl boron-dipyrromethane (BODIPY) dye through the PeT mechanism. However, at the low pH (pH ~ 5 to 6) found in lysosomes, protonation hampers PeT, resulting in a 300 fold increase in photon emission. After conjugation to a trastuzumab antibody, this probe is able to image HER2+ cells after internalization. Because the release of fluorescence is pH dependent, this probe demonstrates reversibility (or ‘‘deactivation’’) when it is ejected from the tumor cell into the more pH neutral extra-cellular environment and when the tumor cell becomes non-viable. This approach is potentially advantageous for protease/enzyme degradation mechanisms where an activated agent that leaks away from the activation site will produce non-specific fluorescence. The pH activated probes will deactivate when they escape their target environment and do not emit fluorescence.

### 4.4. Optical Probes Based on Nanoparticles

**Figure 11:**
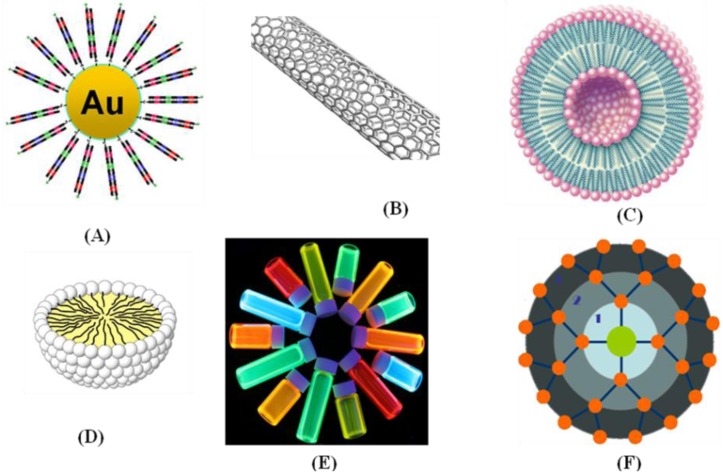
Various types of nanomaterials used as contrast agents for targeted imaging: (A) gold nanoparticle; (B) carbon nanotube; (C) liposome; (D) micelle; (E) quantum dot; and (F) dendrimer.

Nanoparticles provide higher signal intensity than organic dyes, allowing for the detection of targets that are expressed at lower levels [86,87]. In addition, nanoparticles have desirable spectral properties such as flexible excitation bands and tunable fluorescence emission that can be used for multi-spectral imaging. Due to their relatively large surface areas, they are ideal substrates for efficient modification, and can be used to enable multi-valent binding to increase the affinity. There are several promising examples of nanosized materials that have been thoroughly investigated, including quantum dots (QDs), polymeric nanoparticles, carbon nanotubes, and gold nanoparticles, as shown in Figure 11. Over the past decade, there have been significant advances in the field of nanoparticle-based molecular imaging [88,89,90]. An early demonstration of *in vivo* targeted imaging was shown by Gao *et al.* in a mouse model using CdSe-ZnS quantum dots with a co-polymer functionalized surface conjugated to an antibody to target PSMA. *In vivo* targeting studies of human prostate cancer grown in nude mice demonstrated that the QD probes accumulate in tumors by enhanced permeability, retention, and binding to cancer-specific cell surface targets. Multi-color fluorescence imaging of cancer cells *in vivo* has been achieved by subcutaneous and systemic injection of QD-tagged cancer cells [91]. Similarly, quantum dot based imaging probes associated with the RGD peptide (QD705-RGD) as a model targeting system was suggested by Cai *et al*. in an *in vivo* murine xenograft study targeting α_v_β_3_ [92]. While *in vivo* imaging of QDs has been shown to be highly efficacious because of their superior brightness and photostability, the potential for future clinical use is mitigated by concern for long-term toxicities generated by traces of heavy metals. Therefore, metal-free systems would be preferable, provided that they exhibit comparable optical properties.

## 5. Molecular Probes for Scintigraphic Imaging

In clinical research, scintigraphic imaging methods are widely used because of their high sensitivity and compatibility with minute quantities of radio-labeled probes called radiotracers or radiopharmaceuticals. PET and SPECT are the most sensitive imaging techniques in radiation scintigraphy. These modalities are sensitive to systemic concentrations of molecular probes in the sub-picomolar to picomolar range, and can visualize interactions between physiological targets and affinity ligands. Unlike optical fluorophores, there are many radionuclides that can be conjugated to affinity ligands. To develop a new radio-labeled probe, the isotope half-life must be long enough to complete synthesis and to administer to the patient, but no longer than absolutely needed. 

On the other hand, small molecules are cleared rapidly and may require labeling with shorter lived isotopes such as ^11^C and ^18^F, discussed in section 3.2. Another factor in the selection of the ideal radionuclide is the time required to synthesize the compound. To avoid exposure from radiation, the reaction should be performed in a ‘‘hot-cell’’, a radio-protected closed box equipped with an automatic synthesis system. The simple and fast reactions are always most desirable for synthesis of short half-life radionuclides, a task that is often challenging for radiochemists. For PET, a cyclotron can be used to produce the radioisotopes (radioactive chemical elements) that are used to synthesize the radiopharmaceuticals (compounds used to create functional images in the body). E.O. Lawrence first proposed the concept of a cyclotron in 1929 to accelerate charged particles, and then built a cyclotron in 1930 that produced 13-keV H^+^ ions. The cyclotron consists of two hollow semicircular metal electrodes called "dees" because of their shapes. The dees are placed inside an evacuated tank and are connected to a radiofrequency oscillator to supply potential so that their polarities can be alternated, *i.e*., when one dee is positive, the other is negative. The ion source is placed at the center of the gap between the two dees. Ions are normally produced by ionization of an appropriate gas using an electrical arc, such as protons from ionization of H_2_ gas. Each time the ion crosses the dee gap, it gains kinetic energy equal to the product of its charge and the voltage difference between the dees. Finally, as the ions reach the periphery, the beam is removed by an oppositely charged deflector plate and allowed through a window to be used for irradiation of targets. An important area for application of cyclotrons involves the production of short-lived neutron deficient radiotracers for use in positron emission tomography (PET).

Various probes for scintigraphic imaging have been previously presented, and some of them have been successfully applied to clinical research. The glucose analog, ^18^F-fluoro-2-deoxy-d-glucose (^18^F-FDG), has been used to target cells that have high levels of glycolysis. At therapeutic levels, FDG was found to be toxic to the central nervous system and was no longer used clinically [93]. Because of the high sensitivity of PET, ^18^F-FDG can be given safely at very low doses and provide a method for evaluating glucose utilization. Clinically, some success using ^18^F-FDG for estimating the metabolic activity of tumors was achieved, but the ability to distinguish tumor from benign conditions that have upregulated glycolysis was less promising [94,95,96]. This lack of specificity of FDG has led to the development of a new PET tracer, [^18^F]3’-fluoro-L-3’-deoxythymidine (FLT), as a surrogate marker for therapeutic response. FLT phosphorylates in proportion to DNA production, which reflects cell proliferation. FLT offers a faster measure of response than FDG. Although FLT has not been studied as thoroughly as FDG, small-scale trials with n = 10 lung cancer patients have reported an extremely strong correlation (*p* < 0.0001) between the max SUV (maximum pixel standard uptake value) of FLT and Ki-67, an immunohistochemical measure of cell proliferation [97]. In a comparative study with FDG, 26 patients with pulmonary nodules on chest CT were examined with PET by Buck *et al*. using FDG and FLT. Based on the SUV, they reported that the FLT uptake correlates better than FDG for proliferation of lung tumors, and might be more useful as a selective marker for tumor proliferation [98]. In a separate study conducted by Francis *et al*., FLT showed high sensitivity for the detection of extrahepatic disease but poor sensitivity for imaging of colorectal metastases, making it less useful for staging colorectal cancer [99].

Conjugation of radionuclides to antibodies is another approach for developing targeted scintigraphic imaging probes. Several studies have been conducted to demonstrate pre-clinical applications of radio-labeled antibodies for identifying specific cellular targets. Radiolabeled antibodies and antibody derivatives provide the ideal platform for imaging tumor associated cell surface antigens, overcoming the limitations of less specific imaging methods. A number of engineered antibodies, fragments and derivatives have been developed such as ^90^Y conjugated to ibritumomab tiuxetan, a monoclonal antibody to CD20 (clinically approved for treating non-Hodgkins lymphoma), minibodies (~75 kDa) [100], diabodies (~50 kDa) [101], disulfide-stabilized and linear single chain variable fragments (scFv) (~25 kDa) [102], and affibodies (~7 kDa) [103,104]. These engineered antibody fragments have been demonstrated in pre-clinical studies. 

**Figure 12:**
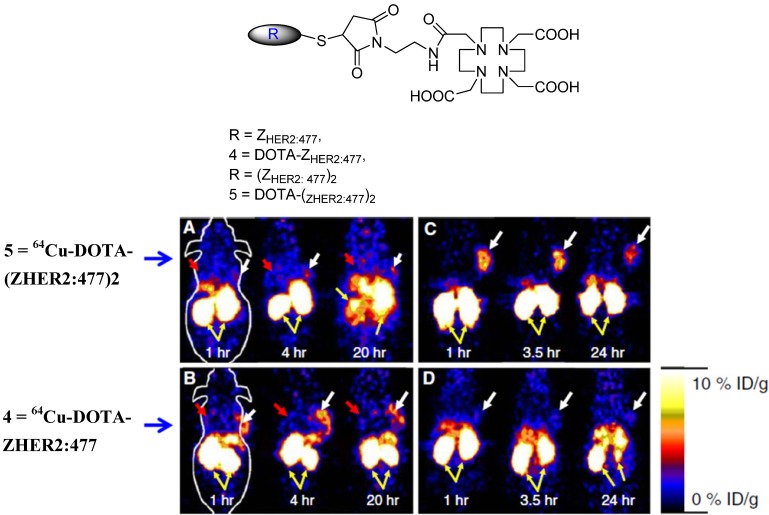
(Top panel) Chemical structures of the anti-HER2 affibody molecules. (Bottom panel) Decay corrected coronal microPET images of nu/nu mice bearing SKOV3 (white arrows) and MDA-MB-435 tumor (red arrows) at 1, 4, and 20 h after tail vein injection of (A) ^64^Cu-DOTA-(ZHER2:477)2 and (B) ^64^Cu-DOTAZHER2:477. Decay corrected coronal microPET images of SKOV3 bearing mice that were pretreated with (C) PBS or (D) 300 μg of Herceptin 48 h before probe administration. Images at 1, 3.5, and 24 h after injection of ^64^Cu-DOTA-ZHER2:477 are shown. Yellow arrows indicate location of kidneys. (Reprinted from [105] with permission.)

In a recent study by Cheng *et al*. human epidermal growth factor receptor type 2 (HER2)-binding affibody molecules in their monomeric and dimeric forms were site specifically modified with the maleimide-functionalized chelator, 1,4,7,10-tetraazacyclododecane-1,4,7-tris(acetic acid)-10-acetate mono (*N*-ethylmaleimide amide) (Mal-DOTA), and radiolabeled with ^64^Cu. Their imaging performance was further evaluated in the SKOV3 tumor mouse model. Biodistribution experiments showed that tumor uptake values of ^64^Cu-DOTA-labeled monomeric and dimeric Her-2 antibodies were 6.12 ± 1.44% and 1.46 ± 0.50% ID/g, respectively, in nude mice (n = 3 each) at 4 h post injection. Moreover, ^64^Cu-labeled monomers exhibited significantly higher tumor/blood ratio than that of radiolabeled dimeric counterpart at all time points examined in this study. As shown in Figure 12, MicroPET imaging of ^64^Cu-DOTA-labeled monomeric Her-2 in SKOV3 tumor mice clearly showed specific tumor localization [105].

Another strategy for performing targeted imaging with a nuclear platform is to use radiolabeled peptide analogs that target endogenous receptors that are over expressed in tumors [106,107]. Most peptide probes are designed to perform a regulatory function and mimic naturally occurring peptides with size ranging from a few to tens of amino acids. These peptides play an important role in modulating physiological conditions via their specific and high-affinity peptide binding receptors. Many of these receptors are highly over expressed in many types of cancer [108]. The growing evidence for peptide-binding receptors over expressed in specific tumors has stimulated interest in the development of peptide-based probes by using radiolabeling techniques [109,110]. Recent advances in combinatorial peptide chemistry and techniques of phage display have led to the development of well established strategies for the design of receptor-specific small peptides [111,112].

Various radionuclides, including ^99m^Tc, ^123^I, ^111^In, ^18^F, ^64^Cu, and ^68^Ga, have been used as labels for peptides via a chelating moiety or a prosthetic group. The peptide can be labeled with the appropriate radiometals using the chelating group covalently conjugated to the peptide or by direct labeling if the functional groups of the peptides are able to act as metal coordinators [113]. The most widely used agents include branched chelators such as di-ethylene tri-amine penta-acetic acid (DTPA), 1,4,7,10-tetra-azacyclododecane-1,4,7,10-tetraacetic acid (DOTA) and their analogs. These chelating agents utilize carboxylate and amine groups to form stable complexes with radioactive metals such as ^111^In, ^64^Cu, ^68^Ga, ^86^Y, ^90^Y, and ^177^Lu. Chelating agents, such as di-amine dithiols, activated mercapto acetyl-glycyl-glycyl-gylcine (MAG3), and hydrazidonicotinamide (HYNIC), are able to chelate metals like ^99m^Tc and ^186^Re. Instead of using chelating agents, a prosthetic group such as *N*-succinimidyl-4-^18^F-fluorobenzoate (^18^F-SFB) is necessary for labeling peptides with ^18^F. 

Various peptides have been developed to utilize these radionuclides, including the FDA approved ^111^In-octreotide and ^99m^Tc-depreotide. ^111^In-octreotide is an 8-mer peptide that was introduced in the mid-1990s. This peptide residue binds to the somatostatin subtype-2 receptor which is over expressed in neuroendocrine tumors, making it an attractive choice for imaging. Depreotide (a somatostatin analog) is another small 10-amino-acid peptide that complexes with ^99m^Tc and binds with high affinity to somatostatin receptor subtypes 2, 3, and 5. These targets are over expressed in SCLC cells and other pulmonary malignancies, as well as in non–small cell lung cancer [114,115]. This compound was approved by the FDA in 2002 and has emerged as a useful non-invasive tool for SPECT imaging to evaluate indeterminate solitary pulmonary nodules and to accurately stage lung cancer. These clinically approved ^111^In labeled DTPA octreotide (OctreoScan) and ^99m^Tc labeled depreotide have proven to be successful and versatile molecular imaging agents [116,117].

Similarly, various other hormone analogs in different stages of pre-clinical and clinical development include bombesin to target the gastrin releasing peptide receptor, vasoactive intestinal peptide (VIP) to target the VIP receptor, and RGD peptide to target α_v_β_3_ integrin [118]. Other peptides undergoing pre-clinical investigation include epidermal growth factor (EGF), glutathione (GSH), and a laminin-ligand (YIGSR). All of these efforts offer promise for localizing different primary and metastatic tumors for producing satisfactory peptide radiopharmaceuticals for diagnostic and therapeutic use.

Another strategy for developing molecular probes for scintigraphic imaging includes the labeling of multi-functional nanoparticles, such as iron oxide, perfluorocarbon, nanotube, quantum dot, micelle, liposome and dendrimer, with suitable radionuclides [119]. 

## 6. Molecular Probes for MRI

Recently, much attention has been given to MRI contrast agents, including both low molecular weight agents and macromolecules, because of their ability to improve image quality. The two major categories of MR probes are paramagnetic and super paramagnetic agents. Paramagnetic metal complexes, such as the gadolinium (III) ion-DTPA (Gd3^+^-DTPA) complex, are widely used in clinical diagnosis. Such gadolinium complexes enhance T1-weighted images by shortening the T1-relaxation time in water. The chelating moiety prevents the paramagnetic lanthanide ion from becoming toxic. Paramagnetic agents generate magnetic moments that speed up the relaxation time of protons in water following a radiofrequency pulse, resulting in shorter T1 and T2 relaxation times and increasing signal. Super paramagnetic agents consist of an iron oxide core or a Fe/Mn composite metal core covered in a polymer matrix to prevent aggregation, and they form a significantly larger magnetic moment than that for paramagnetic agents. Generally, these agents shorten the T2 relaxation times. However, a newer generation of smaller super paramagnetic agents has been reported to affect T1 as well [120]. Both of these agents function primarily through a perfusion mediated process distributed throughout the intravascular and interstitial space. Due to their high vascularity and inefficient lymphatic drainage, they can be used for cancer imaging by localizing tumors and quantifying their size. This approach provides a means to monitor the tumor response to therapy. 

IONPs (iron oxide nanoparticles) are one of the most studied MRI contrast agents because of their superior magnetic properties, ease of modification, and biocompatibility. Currently, several IONP formulations have been approved by the FDA, and are used in the clinic for visualizing the bowel, liver, spleen, and lymph nodes. However, greater sensitivity is needed to image these agents. Several strategies have been pursued to increase sensitivity, including conjugation to peptides and antibodies, approaches that will also perform targeted delivery. So far, this approach has not been widely adopted [121,122]. In order to deliver higher magnetic payloads, Colpitts *et al*. conjugated multiple magnetic nanoparticles on a targeted carrier, such as peptide, dendrimer, and liposome. However, this conjugation step alters the pharmacokinetics of the probe, as well as the magnetic effects of the metal nanoparticles [123]. Several advances in the use of polymeric micelles as MRI contrast agents have recently been reported [124,127], but so far significant enhancements of MR images with solid tumors through accumulation of polymeric micelles have not been obtained. 

## 7. Molecular Probes for US

Targeted contrast-enhanced ultrasound imaging is being recognized as a powerful imaging tool for the detection and quantification of tumors at the molecular level. Microbubbles were initially used to quantify transit times through tissue. Recently, microbubbles have been successfully used for molecular imaging by incorporating ligands on their surface that bind to targets within the microvasculature. These bubbles have also been used for delivery of genes and drugs which can be released locally by disruption of the bubbles with high-energy US. Willmann *et al*. reported integrin-binding knottin peptides conjugated to the surface of microbubbles and demonstrated *in vivo* targeted contrast-enhanced US imaging of tumor angiogenesis. The results suggested that microbubbles conjugated with small peptides provide more signal than those provided by large antibodies [128]. In a separate report by the same group, US imaging with contrast microbubbles was successfully conducted for targeted imaging of VEGFR2 in tumor vessels of a mouse model [129]. There are various kinds of commercially available microbubbles, including (1) Echovist and Levovist from Schering AG; (2) Echogen from Sonus Pharmaceuticals Inc.; (3) Imagent from Alliance Pharmaceuticals Corp.; (4) Definity from Dupont Pharmaceuticals, and (5) Sonazoid from Nycomed Amersham. The limitation on size for clinical microbubbles is determined by the diameter of the pulmonary capillaries, which are the narrowest in the body.

## 8. Dual Labeled Probe for Multi-Modality Imaging

Multi-modality imaging has the potential to overcome the limitations of a single modality and to provide cross-validation by integrating the best features of each approach. This section discusses multi-modal probes that have been designed for one imaging modality but can also function in another. The number of probes that have been developed in this growing field is limited so far. However, we expect that mores probes will be developed as this field matures. The first multi-modality probes were developed [130,132] for imaging in an optical/MRI platform by combining fluorescent dyes with iron oxide nanoparticles. By taking advantage of the dual labels, *in vivo* MR and optical imaging [133,135] could be performed with target-specific delivery via molecular vehicles attached to the probe coating. Other systems include a mono-molecular agents consisting of a heptamethine carbocyanine and an ^111^In-DOTA chelate for optical/scintigraphic and chlorophyll-a analogs conjugated with amino benzyl-DTPA as a photodynamic therapy agent for MRI [136]. 

Kobayashi *et al*. synthesized nanoprobes with multi-modal and multi-color applications which employ a polyamidoamine dendrimer platform (6-PAMAM) linked to both radionuclides and optical probes, permitting dual-modality scintigraphic and 5-color NIR optical imaging of lymphatics in head and neck cancer in a mouse model. Radionuclide imaging provided semi-quantitative information and optical imaging provided qualitative information for each of the 5 lymphatic basins with excellent spatial resolution, suggesting that future applications for sentinel lymph node mapping are promising. Ogawa *et al*. suggested another approach for developing multi-modal probes by combining activatable optical probes with radioactive probes for targeted imaging of Her1 and Her2 tumor bearing mice. ICG dye and ^111^In were selected for the labeling of monoclonal antibodies panitumumab and trastuzumab in Her1 and Her2 expressing tumors in mice. Both conjugates were internalized and showed bright fluorescence only in the target cells on optical imaging, and similar results were obtained with a dual labeled probe in both imaging platforms without affecting the pharmacokinetic properties [137]. Another distinct approach suggested by Liu *et al*. for imaging with the SPECT/MRI platform was based on conjugation of Fe_3_O_4_ nanoparticles and ^125^I with a monoclonal antibody via a PEG linker. The sensitive *γ*-imaging results based on the covalently attached ^125^I provide additional information on the biodistribution of the dual-modality probe, suggesting that this may be a more sensitive approach for evaluating the *in vivo* behavior of nanoparticle-based molecular probes [138].

**Figure 13:**
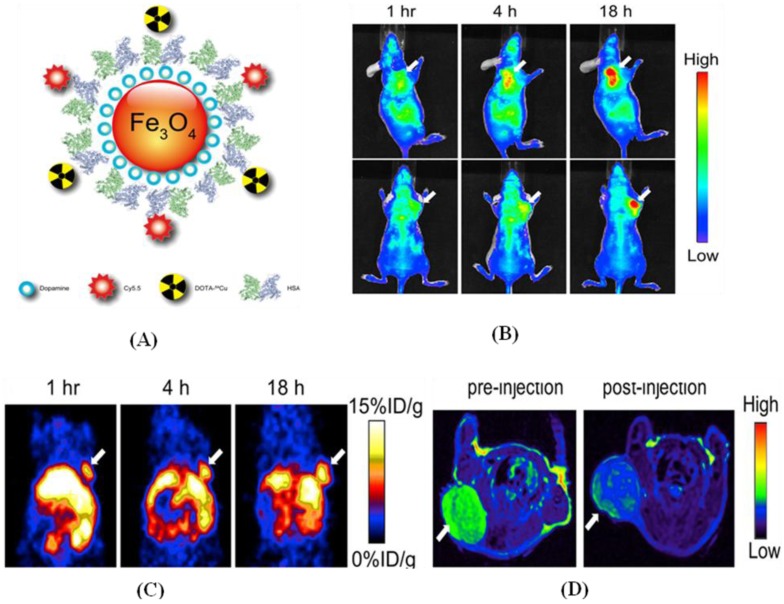
(A) Schematic representation of triple functional probe HSA-IONPs for PET/optical and MR imaging; (B) Representative *in vivo* NIR images of a mouse injected with the probe. Images were acquired 1, 4 and 18 h post-injection; (C) *In vivo* PET images of the mouse are shown after injection at 1, 4 and 18 h; (D) MR images acquired before and 18 h post-injection are shown. (Reprinted from [139] with permission.)

A multi-functional probe for PET/NIR/MRI was recently reported by Xie *et al*. based on dopamine-coated iron oxide nanoparticles. On the surface, human serum albumin (HSA) was encapsulated with dopamine and labeled with Cy5.5 and ^64^Cu-DOTA, and tested in a subcutaneous U87MG xenograft mouse model. *In vivo* PET/NIR/MRI tri-modality imaging was performed, and *ex vivo* analysis with histological examinations was carefully conducted to investigate the *in vivo* behavior of the nanostructures. With the compact HSA coating, the HSA-IONPs demonstrated a prolonged circulation half-life, and showed massive accumulation in the lesions, high extravasation rate, and low uptake of the particles by macrophages at the site of the tumor, as shown in Figure 13 [139].

Kimura *et al*. reported the development of a dual-labeled probe conjugated with ^64^Cu-DOTA and Cy5.5 with a knottin peptide for the imaging of α_v_*β*_3_ and α_v_*β*_5_ integrin receptors. NIR and PET imaging studies in tumor xenograft models showed that Cy5.5 conjugation significantly increased kidney uptake and retention compared to that for the knottin peptide labeled with ^64^Cu-DOTA alone. In the tumor, the dual-labeled ^64^Cu-DOTA/Cy5.5 knottin peptide showed decreased wash-out leading to significantly better retention (*p* < 0.05) compared to that of the ^64^Cu-DOTA-labeled knottin peptide [140].

More recently, Nam Taehwan *et al*. developed tumor targeting chitosan nanoparticles for optical/MR imaging based on polymeric nanoparticle technology. Biocompatible and water-soluble glycol chitosan (MW 50 kDa) was chemically modified with 5-cholanic acid (CA), and conjugated with Cy5.5 and Gd3^+^-DOTA. When the targeted conjugate compound (Cy5.5-GC-Gd^3+^) was systemically administrated into the tail vein of tumor-bearing mice, large amounts of nanoparticles were successfully localized within the tumor, which was confirmed by non-invasive NIR fluorescence and MR imaging simultaneously. These results revealed that the dual-modality imaging probe has the potential to be used as an optical/MR dual imaging agent for cancer treatment [141].

This emerging field of multi-modality imaging using dual labeled probes promises to allow researchers to detect the same probe with multiple imaging techniques. The capability for performing cross-modality validation can provide more accurate and reliable data than with a single imaging modality alone. To date, technical and practical issues associated with dual-labeled probes and multi-modality imaging systems have made it more difficult for translation into clinical applications.

## 9. Summary and Future Prospects

Functional and molecular imaging have become increasingly more directed and specific, taking advantage of rapid technical advances in various imaging modalities and of novel designs for molecular probes in every aspect of cancer. These powerful techniques have provided a meaningful approach to study and image the dynamics of biological processes at the molecular level. Continued progress in our understanding of molecular biology has identified a number of cell surface receptors that can be used as molecular targets. Recent advances brought about by integrating diverse ideas in this inter-disciplinary field have generated novel constructs. In this review, we have presented numerous approaches for the design of exogenous probes that have been developed for their specific applications. Most of the probes have been successfully demonstrated in small animal models, and a few have also reached the clinic. For future applications, we expect that the successes gained from pre-clinical imaging will be translated into the clinic to improving diagnostic accuracy and monitoring of therapy. The characterization of multiple targets, development of new probes, and advancements in imaging instruments promise to lead to more widespread applications. Because a single imaging modality may not be adequate to answer important scientific and clinical questions, multi-modal strategies may become more prominent in the future. Despite these challenges, we must keep in mind that inter-disciplinary research at the interface of imaging science, molecular biology, and probe chemistry will provide numerous opportunities and can lead to the development of novel instruments and new probes for pre-clinical and clinical imaging in the near future. 

## Abbreviations

PET: Positron Emission Tomography
SPECT: Single Photon Emission Computed Tomography
US: Ultrasound
CT: Computed Tomography
OCT: Optical Coherence Tomography
MRI: Magnetic Resonance Imaging
NIR: Near Infra Red
PL: Polylysine
MPEG: Methoxypolyethylene Glycol
FITC: Fluorescein Isothiocyanate
ICG: Indocyanine Green
ITCC: Indotricarbocyanine
AMC: 7-Amino 4-methyl coumarin
ETAR: Endothelial A receptor
VEGFR2: Vascular Endothelial Growth Factor Receptor 2
EGFR: Epidermal Growth Factor Receptor
MMPs: Matrix Metallo Proteinases
GRPR: Gastrin Releasing Peptide Receptor
VCAM-1: Vascular Cell Adhesion Molecule-1
SPARC: Spectral Protein Acidic and Rich in Cystein
HAS: Human Serum Albumin
IONPs: Iron Oxide Nanoparticles
MW: Molecular Weight
CA: Cholenic Acid
BODIPY: Borondipyrromethane
PEG: Polyethylene Glycol
GSH: Glutathione
CdSe-ZnS: Cadmium Selenide-Zinc Sulfide
PDT: Photodynamic Therapy
VIP: Vasoactive Intestinal Peptide
SFB: Succinimidy Fluorobenzote
SCLC: Small Cell Lung Cancer
NSCLC: Non-Small Cell Lung Cancer
HYNIC: Hydrazidonicotinamide
QD: Quantum Dot
IRB: Institutional Review Board
H&E: Hematoxylin and Eosin
TAMRA: Tetramethylrhodamine
FDA: Food and Drug Administration
FDG: Fludeoxyglucose
FLT: Deoxyfluorothymidine
DOTA: 1,4,7,10-tetraazacyclododecane-1,4,7,10-tetraacetic acid
DTPA: Diethylenetriaminepentaacetic acid
PAMAM: Polyaminoamine
